# Supplementary figures and images for: Epidemiology and trends of animal bites in Khoy County, Northwest Iran: A retrospective study (2021–2024)
Source: PLoS One. 2026 Apr 30;21(4):e0348132. doi: 10.1371/journal.pone.0348132 (PMC13132203; doi:10.1371/journal.pone.0348132)

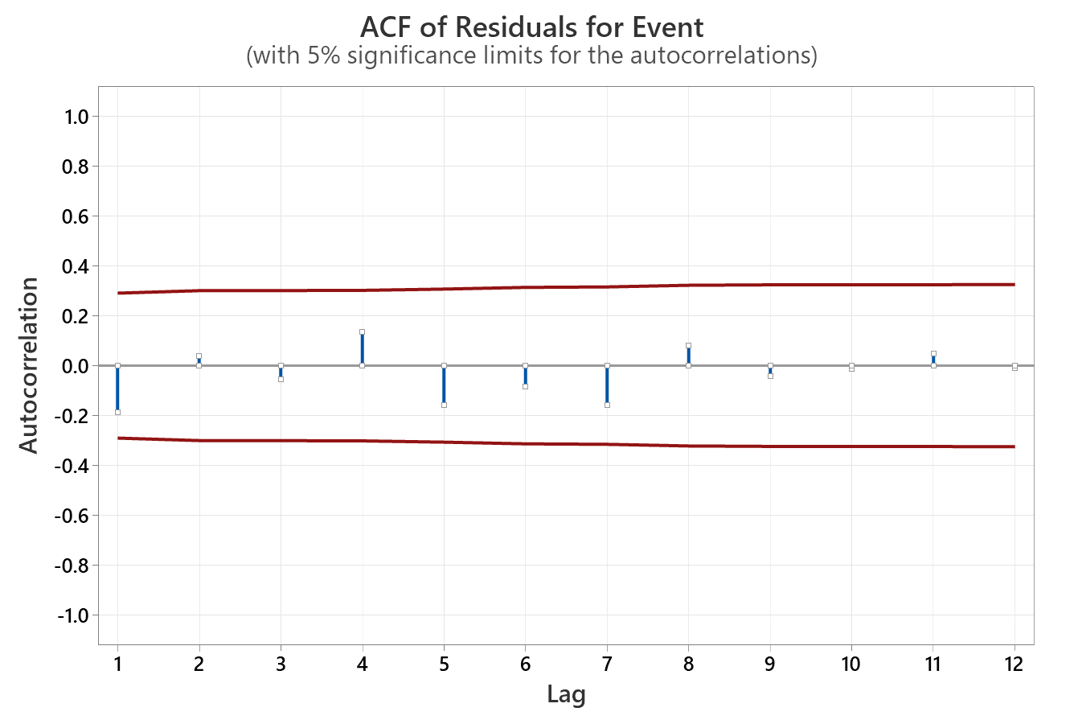

Supplement: S1 Fig — (TIF) [file pone.0348132.s001.tif]

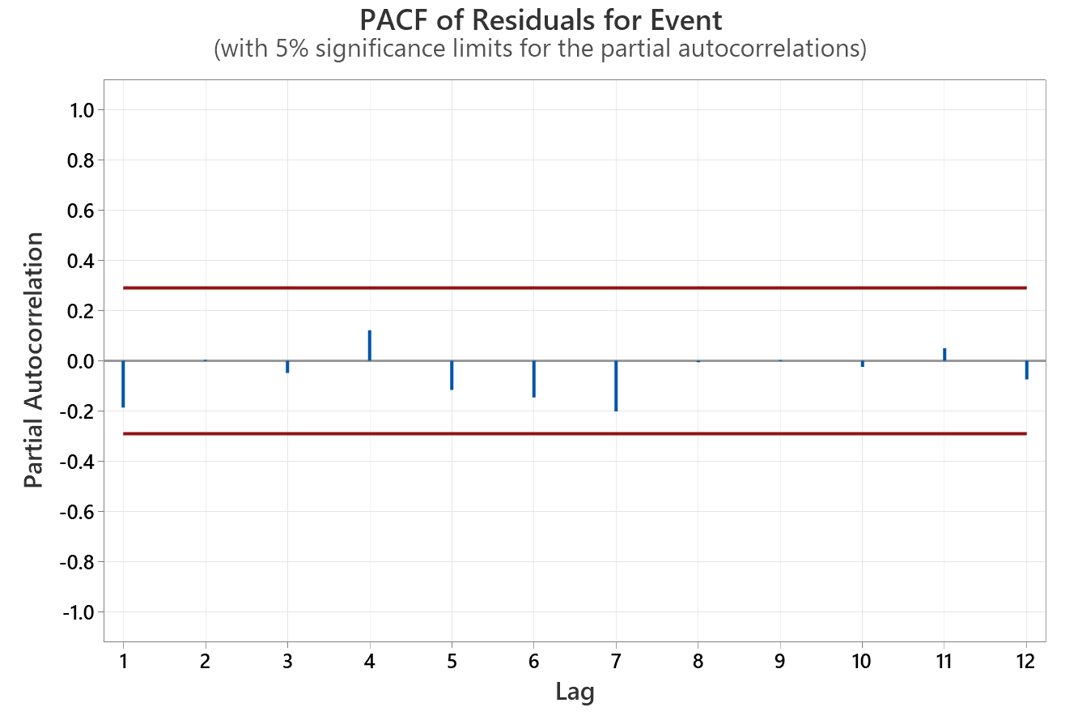

Supplement: S2 Fig — (TIF) [file pone.0348132.s002.tif]

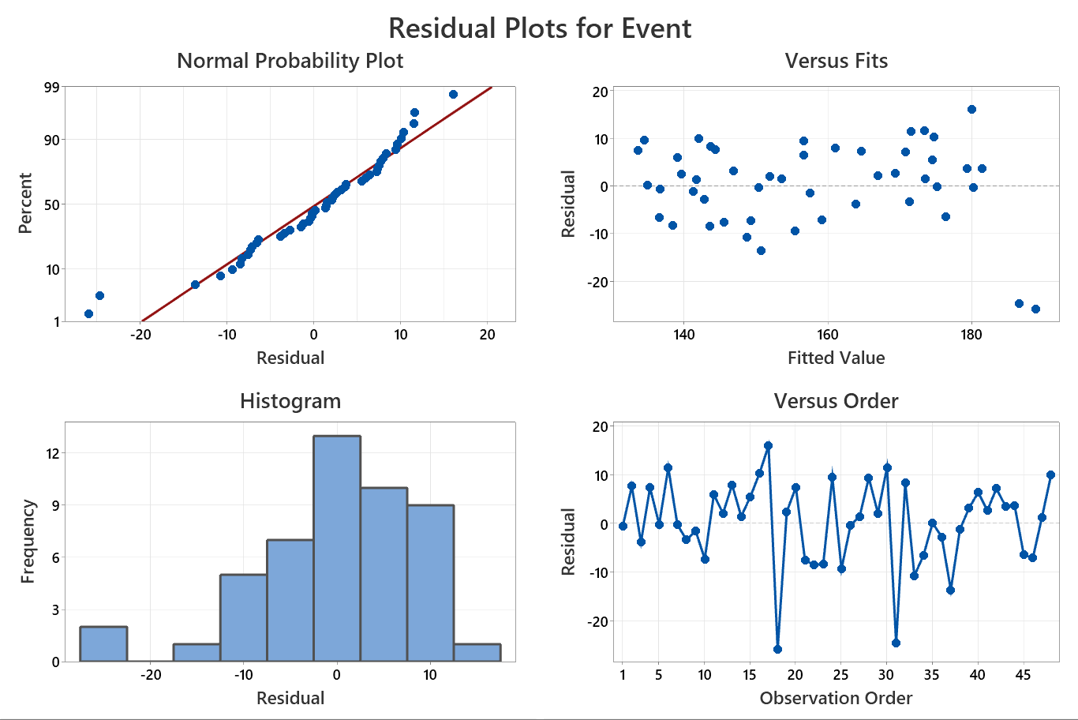

Supplement: S3 Fig — (TIF) [file pone.0348132.s003.tif]
