# Supplementary material for: Epidemiology and trends of animal bites in Khoy County, Northwest Iran: A retrospective study (2021–2024)
Source: PLoS One. 2026 Apr 30;21(4):e0348132. doi: 10.1371/journal.pone.0348132 (PMC13132203; doi:10.1371/journal.pone.0348132)
Supplement: Attachment — Submitted filename: Animal Bites.docx [file pone.0348132.s004.docx]

**Epidemiology and Trends of Animal Bites in Khoy County, Northwest Iran: A Retrospective Study (2021–2024)**

**Parviz Shahmirzalou^1^, Hassan Ebrahimpour Sadagheyani^2,3^**

1. Assistant Professor, Department of Public Health, Khoy University of Medical Sciences, Khoy, Iran. ORCID: 0000-0002-1525-5191, Email: [pshkhoei@gmail.com](mailto:pshkhoei@gmail.com).

2. Associate Professor, Department of Health Information Technology, Khoy University of Medical Sciences, Khoy, Iran. ORCID: 0000-0003-3582-6198, Email: [sadageyani@yahoo.com](mailto:sadageyani@yahoo.com).

3. Associate Professor, Department of Health Information Technology, Neyshabur University of Medical Sciences, Neyshabur, Iran. ORCID: 0000-0003-3582-6198, Email: [sadageyani@yahoo.com](mailto:sadageyani@yahoo.com).

**Corresponding Author**: Hassan Ebrahimpour Sadagheyani, Associate Professor, Department of Health Information Technology, Khoy University of Medical Sciences, Khoy, Iran. ORCID: 0000-0003-3582-6198, Email: [sadageyani@yahoo.com](mailto:sadageyani@yahoo.com).

## Abstract

**Background**

Animal bite is a significant global public health concern because they can lead to rabies, a disease with a very high case fatality. Therefore, this study aimed to describe the demographic and epidemiological characteristics of animal bite cases and to examine temporal trends in their incidence in Khoy County, Northwest Iran, from 21/03/2021 to 19/03/2024.

**Methods**

This cross-sectional descriptive-analytical study included all individuals who were bitten in Khoy County between 21/03/2021 and 19/03/2024, and collected data on demographic characteristics, preventive actions taken after bites, and vaccination status. In addition to descriptive analyses, chi-square tests, trend analysis, time-series regression, and a 12-month forecast were performed.

**Results**

The mean age of bite victims was 23.31 years. The incidence of animal bites per 100,000 population in 2021–2024 was 371.39, 503.23, 506.02, and 397.45, respectively. Dogs accounted for 90% of bite incidents, and the most common circumstances leading to bites were sudden animal attacks, provoking the animal, hunting, and self-defense. Monthly counts revealed a clear 12-month seasonal variation, with the highest incidence occurring in summer and early autumn. The forecasting model predicted that the late spring and summer of 1404 would represent the peak periods of animal-bite incidence.

**Conclusion**

Considering the relatively high incidence of animal bites in Khoy, targeted education of at-risk groups to prevent animal bites and, when bites occur, timely presentation for post-exposure prophylaxis against rabies is recommended.

**Keywords:** Animal Bite, Khoy County, Epidemiology, Incidence, Temporal trend

## Introduction

Animal bites constitute a major public health concern worldwide because, if untreated, they can result in rabies, cause physical injury, and impose substantial healthcare costs(2). Rabies is a lethal viral disease that affects the central nervous system of mammals, including humans; animal bites are a primary route of transmission. Although rabies is vaccine-preventable, it remains a significant public health problem, particularly in areas with limited access to vaccines and post-exposure prophylaxis. Globally, rabies kills tens of thousands of people annually, the majority of whom live in Asia and Africa(3).

According to Iran’s national guidelines for rabies prevention, bites by domestic or wild animals such as dogs, foxes, wolves, jackals, bears, hyenas, cats, bats, raccoons, martens (including species referred to locally as marmot, Indian Grey Mongoose, stoat), ferrets, badgers, and leopards may place a person at risk of rabies; therefore, exposed persons should receive appropriate post-exposure prophylaxis(4). In Iran, the main source of human rabies is animal bites, primarily from dogs and wild animals such as foxes and wolves(5). Given the high case fatality rate associated with clinical rabies, early prevention and control are critical. Rabies is a twin concern in Iran, threatening both public health and agriculture, and the economic burden of treatment expenses and livestock losses highlights the need for epidemiological investigations.

The U.S. Centers for Disease Control and Prevention reports that rabies causes approximately 70,000 deaths annually worldwide and that 99% of human rabies cases outside the United States are attributable to dogs; the principal drivers of rabies are inadequate vaccination coverage and limited access to treatment services in some countries (6). Shakerian and Sadraei, in a systematic review of 33 studies involving 251,000 bites in Iran, reported an average age of 30 among bite victims and a pooled incidence rate of 1,200 bites per 100,000 people. Approximately three-quarters of bite victims were male, and about half occurred in rural areas; most bites involved the lower limbs and were caused mainly by dogs (7).

The incidence of animal bites in Iran varies substantially by geographic location and city. The highest reported incidences (per 100,000 population) include Chaldoran in West Azarbaijan with 541 cases(8), Galikesh in Golestan with 540 cases(9), and Bardsir in Kerman with 433 cases(10). The lowest incidences have been reported in cities such as Sanandaj (67 cases)(11), Bandar Abbas (85 cases)(12), and Kermanshah (114 cases)(13).

Khoy County covers an area of 5,548 km² in northern West Azerbaijan Province (Figure 1) and had a population of 348,664 at the most recent national census in 1395, of whom 198,845 lived in urban areas. ~~The incidence of animal bites in Iran varies substantially by geographic location and city. The highest reported incidences (per 100,000 population) include Chaldoran in West Azarbaijan with 541 cases(8), Galikesh in Golestan with 540 cases(9), and Bardsir in Kerman with 433 cases(10). The lowest incidences have been reported in cities such as Sanandaj (67 cases)(11), Bandar Abbas (85 cases)(12), and Kermanshah (114 cases)(13).~~

~~Khoy County covers an area of 5,548 km² in northern West Azerbaijan Province (Figure 1) and had a population of 348,664 at the most recent national census in 1395, of whom 198,845 lived in urban areas~~. The county is situated within high mountain ranges and experiences a range of climatic conditions, from warm summers to frigid winters. Parts of the county are forested and host diverse flora and wildlife, including brown bears, gazelles, wild sheep, wild boar, hyenas, and raptors such as eagles and falcons (1).

Although animal bites are known to pose a risk of disease, there is limited information on the epidemiology, temporal trends, and geographic distribution of bites in Khoy County. An informal report from the local rabies prevention center in Khoy stated that no confirmed rabies cases had been reported in recent years. Still, a confirmed human rabies case was reported this year. Although national and provincial statistics provide a general overview, county-level studies that incorporate local temporal and spatial characteristics can inform public health interventions more precisely, and relatively few studies have examined demographic and environmental factors that contribute to bite rates over time(7, 14). The lack of such knowledge impacts policymakers, healthcare providers, and local authorities, who need accurate local data to design prevention and education programs to decrease bite risk. Therefore, this study was designed to describe demographic and epidemiologic features of animal bites in Khoy County, western Iran.

## Materials and Methods

This cross-sectional descriptive-analytical study included all individuals who presented to the Animal Bite Prevention Center at Khoy University of Medical Sciences following an animal bite between 21/03/2021 and 19/03/2024. A comprehensive dataset from this registry was accessed and analyzed for the present study on 26/03/2025. According to the national guideline for animal-bite prevention, any contact between domestic or wild animals and humans involving the animal’s teeth and resulting in scratches, bruises, or skin laceration is defined as an animal bite. Animals listed in this guideline include dogs, foxes, wolves, jackals, bears, hyenas, cats, bats, raccoons, martens (marmot, Indian Grey Mongoose, stoat), ferrets, badgers, and leopards(4).

**Data Collection Instrument**

This study was supported and conducted under a research project approved by the Khoy University of Medical Sciences (Ethics Code: IR.KHOY.REC.1404.001: <https://ethics.research.ac.ir/EthicsProposalView.php?&code=IR.KHOY.REC.1404.001>). Data were collected using a researcher-designed checklist based on records from the national Centers for Disease Control and Prevention. The checklist included:

•Demographic characteristics of the victims;

•Vaccination details, including number and timing of doses;

•Serum administration and wound management information;

•Site and nature of the injury;

•Type of animal (domestic/wild, owned/stray, or under observation).

•The authors did not have access to the participants' identifying information.

*Figure 1. Geographical location of Khoy County in West Azerbaijan Province, Iran(1).*

**Occupational Grouping**

•	Group 1 includes children, schoolchildren, students, and housewives.

•	Group 2 includes animal-related jobs, including rangers, farmers, ranchers, keepers of livestock, veterinarians, woodcutters, and shepherds.

•	Group 3: non-animal-related occupations, including military personnel, office workers, laborers, staff of care facilities, janitors, and drivers.

**Classification of Bite Circumstances**

Reasons for animal bites were categorized into four groups:

1.	Sudden animal attack, provoking the animal, hunting, or self-defense;

2.	Feeding, playing with, or caring for an animal;

3.	Contact with a sick or rabid animal;

4.	Other reasons.

**Statistical Analysis**

Both descriptive statistics (measures of central tendency and dispersion, and graphical representations) and inferential statistics (Chi-square or Fisher's exact test, as appropriate) were used for data analysis. Time-series analysis was used to examine the monthly animal bites over time and forecast the number of bites for the next 12 months.

1. Tested for autocorrelation: Correlation across time was assessed using autocorrelation (ACF) and partial autocorrelation (PACF) plots.

2. Tested for stationarity: Mean and variance stationarity were tested for using trend analysis and the Augmented Dickey–Fuller (ADF) test, and when non-stationarity was found, data transformation and differencing were used.

3. Fitted a time-series regression model of the ARIMA or SARIMA family: The difference parameters (d and D) were set to zero initially. The model was fitted after achieving stationarity.

## Results

According to the findings of the present study, a total of 6,553 cases of animal bites were reported in Khoy County from 21/03/2021 to 19/03/2024 - specifically 1,337, 1,840, 1,878, and 1,488 cases in each respective year. The annual incidence rates per 100,000 population were 371.39, 503.23, 506.02, and 397.45 for the years 2021, 2022, 2023, and 2024, respectively. The mean (±SD) age of bite victims was 31.23 ± 18.19 years, 31.15 ± 20.44 years among females, and 31.25 ± 17.57 years among males.

Table 1. The frequency (percentage) of certain demographic characteristics of citizens bitten by animals between 2021 and 2024, and for the whole period studied, together with a proportionality test to verify that the subgroups in the population being studied are assumed to be equal.

*Figure 2. Number of animal bite cases in Khoy County from 2021 to 2024.*

| **Feature** | | **Frequency (%)** | | | | | **Statistic(P)** |
| --- | --- | --- | --- | --- | --- | --- | --- |
|  |  | **2021** | **2022** | **2023** | **2024** | **Total** |  |
| Gender | Female | 155(12%) | 364(20%) | 456(24%) | 364(25%) | 1342(21%) | Z =47.80 (P<0.001) |
|  | Male | 1182 (88%) | 1476(80%) | 1422(76%) | 1124(75%) | 5211(79%) |  |
| Job | Group One | 344 (26%) | 822 (45%) | 899 (48%) | 528 (36%) | 2598 (40%) | Z = -24.31 (P<0.001) |
|  | Group Two | 741 (55%) | 316 (17%) | 243 (13%) | 327 (22%) | 1629(25%) |  |
|  | Group Three | 245 (18%) | 689 (37%) | 700 (37%) | 631 (42%) | 2268(35%) |  |
|  | Missing | 7(1%) | 13(1%) | 36(2%) | 2(0%) | 58 (1%) |  |
| Residence Location | Village | 1033(77%) | 986(54%) | 984(52%) | 704(47%) | 3708(57%) | Z = -11.72 (P<0.001) |
|  | City | 296(22%) | 821(45%) | 888(47%) | 751(51%) | 2765(42%) |  |
|  | Missing | 8(1%) | 33(2%) | 6(0%) | 33(2%) | 80(1%) |  |
| Cause of Animal Bite | Reason 1 | 1100(82%) | 1350(73%) | 1390(74%) | 982(66%) | 4822(74%) | Z = -78.55 (P<0.001) |
|  | Reason 2 | 197(15%) | 449(24%) | 461(25%) | 235(16%) | 1342(21%) |  |
|  | Reason 3 | 2(0%) | 11(1%) | 18(1%) | 233(16%) | 264(4%) |  |
|  | Reason 4 | 37(3%) | 28(2%) | 8(0%) | 18(1%) | 91(1%) |  |
|  | Missing | 1(0%) | 2(0%) | 1(0%) | 20(1%) | 24(1%) |  |
| Animal Type | Dog | 1333(100%) | 1826(99%) | 1866(99%) | 935(63%) | 5964(90%) | Z = -80.14 (P<0.001) |
|  | Cat | 3(0%) | 12(1%) | 6(0%) | 472(32%) | 499(8%) |  |
|  | Goat and Sheep | 0(0%) | 0(0%) | 0(0%) | 34(2%) | 34(1%) |  |
|  | Other | 0(0%) | 0(0%) | 0(0%) | 25(2%) | 25 (0%) |  |
|  | Missing | 1(0%) | 2(0%) | 6(0%) | 22(2%) | 31(1%) |  |
| Before Animal Status | Domestic | 1229(92%) | 1506(82%) | 1614(86%) | 1180(79%) | 5538(85%) | Z = -64.93 (P<0.001) |
|  | Wild | 2(0%) | 8(0%) | 10(1%) | 12(1%) | 32(1%) |  |
|  | Stray | 6(0%) | 169(9%) | 100(5%) | 232(16%) | 508(8%) |  |
|  | Missing | 100 (8%) | 157 (9%) | 154 (8%) | 64 (4%) | 475 (7%) |  |
| After Animal Status | Owned or supervised | 1207(90%) | 1458(79%) | 1433(76%) | 704(47%) | 4808(73%) | Z = -73.77 (P<0.001) |
|  | Escaped | 114(9%) | 242(13%) | 218(12%) | 230(16%) | 805(12%) |  |
|  | Killed | 4(0%) | 16(1%) | 21(1%) | 17(1%) | 58(1%) |  |
|  | Missing | 12(1%) | 124(7%) | 206(11%) | 537(36%) | 882(14%) |  |
| Antibiotic Prescription | No | 1253(94%) | 1701(92%) | 1797(96%) | 1437(97%) | 6198(95%) | Z = -72.22 (P<0.001) |
|  | Yes | 84(6%) | 139(8%) | 79(4%) | 51(3%) | 353(5%) |  |
|  | Missing | 0(0%) | 0(0%) | 2(0%) | 0(0%) | 2(0%) |  |
| Vaccine Type | PCEC | 1(0%) | 2(0%) | 278(15%) | 43(3%) | 324(5%) | Z = 72.63 (P<0.001) |
|  | VERO | 1335(100%) | 1835(100%) | 1595(85%) | 1441(97%) | 6216(95%) |  |
|  | Missing | 1(0%) | 3(0%) | 5(0%) | 4(0%) | 13(0%) |  |
| Serum Therapy | No | 1256(94%) | 1590(86%) | 1634(87%) | 1359(91%) | 5849(89%) | Z = -63.57 (P<0.001) |
|  | Yes | 81(6%) | 250(14%) | 243(13%) | 129(9%) | 703(10%) |  |
|  | Missing | 0(0%) | 0(0%) | 0(0%) | 0(0%) | 1(0%) |  |
| Incident Location | At Home or Work | 1330(100%) | 1820(99%) | 1849(99%) | 1472(99%) | 6471(99%) | Z = 43.06 (P<0.001) |
|  | While traveling | 7(0%) | 16(1%) | 24(1%) | 12(1%) | 59(1%) |  |
|  | Missing | 0(0%) | 4(0%) | 5(0%) | 4(0%) | 13(0%) |  |
| Incident Area | Village | 1074(80%) | 1082(59%) | 1046(56%) | 744(50%) | 3947(60%) | Z = -18.13 (P<0.001) |
|  | City | 247 (19%) | 709 (39%) | 804 (43%) | 723 (49%) | 2492(38%) |  |
|  | Missing | 16(1%) | 49(3%) | 28(2%) | 21(1%) | 114(2%) |  |
| Bite by Animal Owner | No | 399(30%) | 979(53%) | 1366(73%) | 1074(72%) | 3818(58%) | Z = -13.99 (P<0.001) |
|  | Yes | 937(70%) | 859(47%) | 508(27%) | 394(27%) | 2698(41%) |  |
|  | Missing | 1(0%) | 2(0%) | 4(0%) | 20(1%) | 27(0%) |  |

**Post-exposure Prophylaxis and Wound Management**

Between 2021 and 2024, 98%, 97%, 96%, and 97% of bite victims, respectively, sought timely post-exposure prophylaxis (PEP). Overall, 12% received two vaccine doses, 86% received three doses, and 3% received four doses. In 98% of all cases, the wound site was washed with soap and water immediately after exposure. The number of male victims was consistently higher than that of females, and this difference was statistically significant (P < 0.001). But the proportion of male and female victims did not vary significantly over the years studied. Group 1 (children, students, university students, and housewives) had the highest proportion of bite cases between jobs, and the proportion among occupational categories was significantly different (P < 0.001).

The maximum number of bites was in:

•Group 2 in 2021,

•Group 1 in 2022 and 2023,

•Group 3 in 2024. In 2021,

Almost 77% of bites occurred in rural areas, whereas in 2024, 51% were reported in urban areas. The most common causes were animal attack, provocation, hunting, defense, feeding, playing with, or caring for animals.

**Cause and Type of Animals Involved**

The most common causes of bites were sudden animal attacks, provocation, hunting, defense, feeding, playing with, or caring for animals. Throughout the study period, dogs and domestic animals were the predominant sources of bites, and 71% of the biting animals were owned or under observation. Antibiotics were prescribed in 5% of cases, and anti-rabies serum was administered in 10%.

**Temporal Distribution**

The lowest monthly numbers of bites during 2021-2024 occurred in:

•	Farvardin (March–April) – 136 cases,

•	Bahman (January–February) – 130 cases,

•	Dey (December–January) – 130 cases,

•	Azar (November–December) – 134 cases.

The highest numbers were recorded in:

- Shahrivar (August–September) – 183 cases,
- Mordad (July–August) – 196 cases,
- Shahrivar (August–September) – 185 cases,
- Mehr (September–October) – 185 cases (Figure 2).

***Seasonal Pattern and Trend Analysis***

***Figure 2:*** The number of animal bites was seasonal, with a higher number during the first half of the year (spring and summer) and a lower number during the second half (autumn and early winter). The number of animal bites reached its maximum in Mordad 2022 (July–August) and its minimum in Esfand 2022 (February–March) and Bahman 2023 (January–February).

*Figure 2. Number of animal bite cases in Khoy County from 2021 to 2024.*

**Figure 3**: The average number of bites per month shows that mid-year months had the highest incidence rates.

*Figure 3. Monthly incidence of animal bites, along with 95% confidence interval for the mean animal bites in that month.*

**Figure 4**: Both the autocorrelation and partial autocorrelation plots showed that the first lag was significant, meaning that the number of bites in a given month was significantly related to the number of bites in the previous month.

Subsequent trend analysis yielded the following regression equation:

Equation 1: "Number of bite cases"=162.92-0.25×"Time period"

The regression coefficient (–0.25) was not statistically significant (P = 0.186), as shown in Equation (1), which showed no significant linear trend in the monthly frequency of bites. The Augmented Dickey–Fuller (ADF) test statistic was “–3.55” (P = 0.007), indicating the data were stationary.

*Figure 4. Autocorrelation (right) and partial autocorrelation (left) graphs of the number of animal bites during the years 2021 to 2024.*

**Time-Series Model and Forecast**

Since the mean and variance were stationary, time-series modeling was conducted. The final fitted model was SARIMA (2,0,0)(1,0,0)₁₂, with an autoregressive component of order 3, a first-order seasonal autoregressive component, and a 12-month seasonal period. The model parameters are shown in Table 2. According to Equation (1), the regression coefficient (–0.25) was not statistically significant (**p=0.186**), indicating that no meaningful linear trend existed in the monthly frequency of bites.

Table 2. The final model for the time series of animal bites in Khoy County.

| **Parameter** | | **Beta** | **t (P-value)** |
| --- | --- | --- | --- |
| Constants | | 59.5 | 60.65 (<0.001) |
| Autocorrelation | 1^st^ order | 1.19 | 7.96 (0.001) |
|  | 2^nd^ order | -0.30 | -1.30 (0.200) |
|  | 3^rd^ order | -0.27 | -1.77 (0.083) |
| Seasonal Moving average-12 | | 0.23 | 1.29 (0.202) |

The Box–Pierce (Ljung–Box) chi-square test for lags 12, 24, and 36 was not significant (P = 0.497, 0.413, 0.610), indicating no autocorrelation among residuals. Based on this final model, the monthly number of animal-bite cases for the subsequent 12 months was predicted (Table 2).

*Figure 5. The number of animal bites in Khoy County in the next 12 months.*

The predicted number of cases is expected to increase initially, decrease, and then increase again toward the end of the forecast year (see Figure 5), reflecting a seasonal pattern.
Discussion

The present study aimed to investigate the epidemiological characteristics and temporal variations of animal-bite incidence in Khoy County between 2021 and 2024. Most bite victims are male, residents of rural areas, and belong to occupational group 1, which includes children, students, university students, and housewives.

Similar to the present findings, Rostampour (15), Khan(16), Kiakalayeh(17) , and Varisli(18) also reported that the majority of bite victims were male. In contrast, a study conducted in Greece found that 41% of dog-bite victims were female (19), which is considerably higher than in our study (21%). Mirzaei et al.(20), in East Azerbaijan Province, found that most victims were workers, farmers, or livestock breeders, whereas Alian et al.(21) reported that “self-employed” individuals were most frequently affected.

In the current study, the site of bite varied but showed differences from previous findings.
For example, Khan et al.(16) reported that 70% of bites in children and 86% in adults occurred on the lower extremities. In contrast, the present study found these proportions to be 46% and 38%, respectively, indicating a lower rate of leg injuries. The mean age of bite victims in our study was 31 years, with the 10–19 and 40–49 age groups most frequently affected.
Rostampour(15) reported that the 19–59-year age group (62%) was most affected; Kiakalayeh (17) found the “more than 50” years group (24%) dominant, and Khan et al.(16) observed that 61% of victims were over 18 years old. Similarly, Dougas(19) reported that individuals aged 50–59 years had the highest incidence, while Varisli(18) found mean ages of 22 years for men and 26 years for women.

Regarding the circumstances of bites, the most common reasons were provoking the animal, sudden attacks, or self-defense. Although dogs were responsible for the vast majority (90%) of bite cases—mainly due to their proximity to humans—further analysis showed that cat bites had increased sharply in recent years, rising from 0% in 2021 to 32% in 2024.
This increase may reflect a growing population of stray cats and dogs in urban areas. In most cases, the attacking animal was owned or under observation, and in 10–47% of these cases, the animal bit its own owner. Overall, approximately 60% of animal-bite cases occurred in urban areas, similar to the report by Varisli (18) In a study conducted in Isfahan, Abbaspour et al. (22) reported that the leading cause of bites was sudden animal attack (49%). Among males, the most common reason was sudden attack (52%), while among females it was self-defense (17%).

In our study, most victims received the VERO cell vaccine, and in nearly 90% of cases, neither antibiotics nor anti-rabies serum were required. Nikbakht et al.(23) Similarly, it was reported that 87% of animal-bite victims in Mazandaran Province received the VERO vaccine. Geographically, most bites occurred at or near the victim’s residence or workplace, predominantly in rural areas—consistent with Nikbakht et al. (23), who reported that 92% of bites occurred in residential or work settings, with half of these instances taking place in urban Mazandaran. Alian et al.(21) found that 57% of bites occurred in rural areas, whereas Khan et al.(16) reported that more than three-quarters of dog bites occurred on streets, and only 6% took place at home.

In the current study, less than 4% of victims delayed post-exposure prophylaxis (PEP).
Overall, 86% completed three vaccine doses. Rostampour et al. (15) found a 36% delay in PEP in West Azerbaijan Province between 1391 and 1397, with 77% of victims receiving three doses. Varisli et al.(18) reported a 2% delay, with 25% receiving three doses, 21% four doses, and 33% five doses of the rabies vaccine. Follow-up of animals after biting events in the current study showed that the outcome was unknown in only 14% of cases, compared with 17.5% in Rostampour et al.(15).

The incidence of animal bites remains a major concern, and preventive measures are crucial.
In the present study, the annual incidence rates per 100,000 population were 371.39, 503.23, 506.02, and 397.45, respectively. In the systematic review by Shakerian and Sedraei(7), which included 33 studies, the lowest reported incidence was 67 (Mohammadi et al.(11)) and the highest 541 (Babazadeh et al. (8)). Accordingly, the incidence rate in Khoy County was higher than in most Iranian cities. Shakerian and Sedraei (7) also reported a pooled national incidence of 1,200 (!) cases per 100,000 population using meta-analysis, highlighting the substantial burden of this issue. Kiakalayeh et al.(5) reported animal-bite incidence rates in Gilan Province between 1395 and 2022. For 2021, the incidence was 358.3 per 100,000, compared with 371.39 in our study, showing that the rate in Khoy was higher. Across all four years (2021–2024), the incidence in Khoy exceeded that reported for Gilan over six years (1395–2021).

The present study conducted a detailed time-series analysis, including testing for mean and variance stationarity, trend analysis, autocorrelation, and partial autocorrelation, and evaluation of 48 model variants. Ultimately, a seasonal model with a 12-month periodicity was selected to forecast animal-bite cases for the next year. Mirzaei et al.(20) performed a similar analysis in East Azerbaijan Province; however, their Figure 3 shows non-stationarity in variance, indicating that variance increased over time, whereas in our study, appropriate transformations achieved stationarity before model fitting.

Some studies, such as Khan et al.(16), did not assess the temporal trend of animal bites. Tuckel and Milczarski (2020)(24) analyzed the time series of dog-bite incidence in the United States from 2005 to 2018. Their results showed that children under nine years old had the highest bite rates throughout the study period. In contrast, in our study, the 10–19 and 40–49 age groups had the highest incidence. In the U.S. data, incidence trends fluctuated (decreasing–increasing–decreasing–increasing–decreasing from 2016 to 2018. Although the time frames differ, our study revealed an increasing trend from 2021 to 2022, followed by a decline in 2024(24). Furthermore, throughout the study period, the incidence rate in Khoy was more than twice that reported in the United States.

## Conclusion

The annual incidence of animal bites in Khoy County was found to be higher than the national average and greater than that reported in many neighboring cities. Men, children, students, university students, and housewives were at higher risk of animal bites. Contrary to the common belief that bites occur mainly in rural areas, the study revealed an increasing and concerning trend among urban residents. Dogs accounted for approximately 90% of all bite cases. Given this and the observed urban increase, continuous and well-structured educational and preventive interventions are urgently required in Khoy County. Although treatment and prophylactic procedures for animal bites and rabies are being implemented carefully and promptly—ensuring that all bite victims in the county receive appropriate medical care and prophylaxis according to the national protocol—further preventive measures are necessary to reduce bite incidence.

The temporal trend analysis showed that the peak of animal bites occurs during summer and early autumn. Therefore, educational campaigns and preventive programs should be intensified during these months. Through time-series analysis, correlations, trend patterns, and regression models of bite incidence were identified. The model predicts that in the upcoming 12 months, the incidence of animal bites will increase until summer, then decrease from autumn through late winter, and that this pattern is likely to persist in future years.

Given the adequate availability of vaccines and related prophylactic resources, it is recommended that **policy-makers and health managers** prioritize strategies aimed at reducing the incidence of animal bites in this county. Such efforts, particularly through **continuous education of high-risk groups**, will not only lower vaccine and antibiotic consumption but also reduce human resource demand—ultimately improving **efficiency** and achieving **cost savings** in public health programs.

**Acknowledgements**

The authors sincerely thank all colleagues from the Health and Research Deputy Offices of Khoy University of Medical Sciences who provided invaluable assistance throughout the study.

**References**

1.	Getting to know Khoy County. Khoy County Governorship: Khoy Governorate; [updated Retrieved November 4, 2024. Available from: <https://www.khoy-ag.ir/fa/wagov/aboutkhoy>.

2.	Williams BM, Purcell L, Kayange L, Gallaher J, Charles A. Characteristic and outcomes of human and animal bites in Malawi. Injury. 2021;52(8):2188-93.

3.	Organization WH. Rabies: World Health Organization; 2024 [Available from: <https://www.who.int/news-room/fact-sheets/detail/rabies#:~:text=Rabies%20is%20a%20vaccine%2Dpreventable,%2C%20cats%2C%20livestock%20and%20wildlife>.

4.	Shirzadi MR, Bashar R, Farahtaj F, Pourmozafari J, Amiri B, Zeinali M. National guidelines for controlling animal bites and rabies in humans: Ministry of Health and Medical Education of Iran; 2022.

5.	Davoudi Kiakalayeh A GZ, Mohammadi R, Kanafi Vahed L, Davoudi-Kiakalayeh S. Trends in Animal Bites and Rabies-related Deaths in Northern Iran: Implications for Public Health Interventions. Archives of Iranian medicine. 2024;27(5):272-6.

6.	Global Rabies: What You Should Know USA: Center for Disease Control and prevention; 2024 [Available from: <https://www.cdc.gov/rabies/around-world/index.html>.

7.	Shakerian S, Sadraei M. Epidemiology of animal bite in Iran: A Systematic review and meta-analysis. Journal of Research in Medical Sciences. 2023;28(1):59.

8.	Babazadeh T, Nikbakhat HA, Daemi A, Yegane-kasgari M, Ghaffari-fam S, Banaye-Jeddi M. Epidemiology of acute animal bite and the direct cost of rabies vaccination. Journal of Acute Disease. 2016;5(6):488-92.

9.	Maleka A, Behnampour N, Mirkarimi SK, Khosravi S, Khosravi A. The epidemiologic status of animal bite and the effect of wasting stray dogs on the incidence of animal bites in Galikesh County since 2009 until 2013. Jorjani Biomedicine Journal. 2017;5(2):91-6.

10.	Hesseini S, Baneshi MR, Khajeh Kazemi R, Mashayekhi M, Gharaei Khezripour Y, Zolala F. Geographical Distribution, Time Trend, and Epidemiological Characteristics of Animal-Bite Cases in Bardsir, 2010-2014. Journal of Community Health Research. 2017;6(4):216-22.

11.	mohammadi s, mohammadi n, karimi m, Afkhamzadeh AR. the Investigate frequency of animal bites in the human population all urban and rural areas of Sanandaj city in the period of 2008-2014. Zanko Journal of Medical Sciences. 2017;18(56):78-87.

12.	Nodez SMM KM, Khosravi M, Fekri S, Jaberhashemi SA. Epidemiological outline of animal bites in Bandar Abbas County,

Iran. International Journal of Zoology Studies. 2017;2:242-6.

13.	Ghasemi M HZ, Bagherabadi S. Animal Bites in the City of Kermanshah. 2011‑2015. Iranian Journal of Infectious Diseases. 2016;21:39-45.

14.	Gaffari-fam S, Sarbazi E, Moradpour H, Soleimanpour H, Azizi H, Heidari S. Epidemiological Patterns, Trends of Animal Bites and Factors Associated with Delays in Initiating Post-Exposure Prophylaxis for Rabies Prevention in Hurand, Iran: A Cross-Sectional Study. Journal of Clinical and Basic Research. 2021;5(2):48-56.

15.	Rostampour F, Khalkhali HR, Masudi S. Prophylactic Measures Implemented for Cases of Animal Bite in West Azerbaijan Province from 2012 to 2019. Iranian Journal of Epidemiology. 2023;19(3):207-16.

16.	Khan UR, Soomar SM, Ghazi SM, Naeem R, Kerai S, Jamali S. Epidemiological pattern and management of dog bite injuries in Karachi, Pakistan: A cross-sectional study. Injury. 2023;54 Suppl 4:110473.

17.	Ali DK, Zakiye G, Reza M, Leila Kv, Sajad D-K. Trends in Animal Bites and Rabies-related Deaths in Northern Iran: Implications for Public Health Interventions. Archives of Iranian Medicine. 2024;27(5):272-6.

18.	Varisli B, Yildirim, S., Celik, H., Bulut, M., & OCAK, Ö. Assessment of Emergency Service Attendance Due to Rabies Suspect Animal Bites in the Van Region. EURASIAN JOURNAL OF EMERGENCY MEDICINE. 2019;18(4):197-202.

19.	Dougas G, Katerelos P, Chatzianastasiou S, Mellou K. The epidemiology of dog bite injuries in Greece: preliminary results of a surveillance scheme. Hippokratia. 2023;27(3):82-8.

20.	Mirzaei B, Doshmangir L, Khabiri R, Teimouri H, Razzaghi A, Sergeevich Gordeev V. Incidence and forecasting of animal bites and trends in post-exposure prophylaxis (PEP) consumption in East Azerbaijan province. Journal of Zoonotic Diseases. 2025;9(3):874-86.

21.	Alian D, Namayande M, Ghaneian Mt, Ehrampoosh Mh, Madadizadeh F. Trend of animal bites and it's related ecological factors in Jahrom county during 2014-2018. Pars Journal of Medical Sciences. 2022;18(4):35-44.

22.	Abaspoor Najafabadi R, Hajari A, Fadaei Nobari R, Ramezanpoor J, Nasirian M. Epidemiological Study of Animal Bites and Related Factors in Urban and Rural Population under the Auspices of Isfahan University of Medical Sciences in 2019. Military Caring Sciences. 2023;10(4):350-61.

23.	Nikbakht R, Alian S, Eslami S, Karbasi A, Sobhanian P. Trend of Animal Bites and Challenges Related to Community Health inMazandaran: A Retrospective Descriptive Study. Journal of health research in community. 2025;11(1):1-10.

24.	Tuckel PS, Milczarski W. The changing epidemiology of dog bite injuries in the United States, 2005–2018. Injury Epidemiology. 2020;7(1):57.
