# Supplementary material for: Epidemiology and trends of animal bites in Khoy County, Northwest Iran: A retrospective study (2021–2024)
Source: PLoS One. 2026 Apr 30;21(4):e0348132. doi: 10.1371/journal.pone.0348132 (PMC13132203; doi:10.1371/journal.pone.0348132)
Supplement: Attachment — Submitted filename: 03-Response to Reviewers..docx [file pone.0348132.s007.docx]

PONE-D-25-60245

Epidemiology and Trends of Animal Bites in Khoy County, Northwest Iran: A Retrospective Study (2021–2024)

**Reviewer's Responses to Questions**

Comments to the Author

1. Is the manuscript technically sound, and do the data support the conclusions?

The manuscript must describe a technically sound piece of scientific research with data that supports the conclusions. Experiments must have been conducted rigorously, with appropriate controls, replication, and sample sizes. The conclusions must be drawn appropriately based on the data presented.

Reviewer #1: Yes

Authors' Response: Thank you very much.

2. Has the statistical analysis been performed appropriately and rigorously?

Reviewer #1: Yes

Authors' Response: Thank you very much.

3. Have the authors made all data underlying the findings in their manuscript fully available?

The PLOS Data policy requires authors to make all data underlying the findings described in their manuscript fully available without restriction, with rare exception (please refer to the Data Availability Statement in the manuscript PDF file). The data should be provided as part of the manuscript or its supporting information, or deposited to a public repository. For example, in addition to summary statistics, the data points behind means, medians and variance measures should be available. If there are restrictions on publicly sharing data—e.g. participant privacy or use of data from a third party—those must be specified.

Reviewer #1: Yes

The data underlying this study are owned by Khoy University of Medical Sciences (Khoy, Iran) and contain potentially sensitive health information. Due to institutional data ownership policies and privacy considerations, the dataset cannot be made publicly available. Qualified researchers may request access to anonymized and de-identified data. Data requests will be reviewed by the Research Committee of Khoy University of Medical Sciences.

Requests should be directed to:
Dr. Mobin Sokhanvar
Head of the Research Committee
Khoy University of Medical Sciences
Email: Sokhanvar_m@khoyums.ac.ir

4. Is the manuscript presented in an intelligible fashion and written in standard English?

PLOS ONE does not copyedit accepted manuscripts, so the language in submitted articles must be clear, correct, and unambiguous. Any typographical or grammatical errors should be corrected at revision, so please note any specific errors here.

Reviewer #1: No

Authors' Response: Manuscript reviewed and corrected for unclear, typographical, or grammatical errors.

5. Review Comments to the Author

Please use the space provided to explain your answers to the questions above. You may also include additional comments for the author, including concerns about dual publication, research ethics, or publication ethics. (Please upload your review as an attachment if it exceeds 20,000 characters)

Reviewer #1: study is good but some of revisions are required as following (Detailed comments are in manuscript)

1. Language and structural issues - need major revision in grammar, remove duplication of sentences and inconsistency.

Authors' Response: Manuscript reviewed and corrected for unclear, typographical, or grammatical errors.

2. Introduction: duplication of sentences- require editing.

Authors' Response: Thank you. You're right. Duplicate sentences identified and corrected.

3. Methodology: need clarification and elaboration for study design, data primary or secondary, inclusion criteria, statistical software used.

Authors' Response: The material and Methods of the manuscript were surveyed again, and regarding the dataset, inclusion criteria, and software, new explanations were added.

4. Results: Describe the results coherently and in flow of tables. table 1 need to reorganize, calculated % need to check, duplication of variables.

Authors' Response: Table 1,2 in the result section corrected. In particular, the percentages of subgroups of given variables are controlled.

5. Discussion: could be better and refinement requited.

Authors' Response: A few correction have been done.

6. conclusion: could be shorter and specific

Authors' Response: done.

7. References: need proper style formatting in all references.

Authors' Response: All references were rechecked, duplicate references were removed, and references were checked for compliance with the Vancouver style. Now, there are 24 references.

6. PLOS authors have the option to publish the peer review history of their article (what does this mean?). If published, this will include your full peer review and any attached files.

If you choose “no”, your identity will remain anonymous but your review may still be made public.

Do you want your identity to be public for this peer review? For information about this choice, including consent withdrawal, please see our Privacy Policy.

Reviewer #1: No

[NOTE: If reviewer comments were submitted as an attachment file, they will be attached to this email and accessible via the submission site. Please log into your account, locate the manuscript record, and check for the action link "View Attachments". If this link does not appear, there are no attachment files.]

To ensure your figures meet our technical requirements, please review our figure guidelines: https://journals.plos.org/plosone/s/figures

You may also use PLOS’s free figure tool, NAAS, to help you prepare publication quality figures: https://journals.plos.org/plosone/s/figures#loc-tools-for-figure-preparation.

NAAS will assess whether your figures meet our technical requirements by comparing each figure against our figure specifications.

Authors' Response: No.

4. We note that Figure 1 in your submission contain map images which may be copyrighted. All PLOS content is published under the Creative Commons Attribution License (CC BY 4.0), which means that the manuscript, images, and Supporting Information files will be freely available online, and any third party is permitted to access, download, copy, distribute, and use these materials in any way, even commercially, with proper attribution. For these reasons, we cannot publish previously copyrighted maps or satellite images created using proprietary data, such as Google software (Google Maps, Street View, and Earth). For more information, see our copyright guidelines: http://journals.plos.org/plosone/s/licenses-and-copyright.

We require you to either (1) present written permission from the copyright holder to publish these figures specifically under the CC BY 4.0 license, or (2) remove the figures from your submission:

1. You may seek permission from the original copyright holder of Figure 1 to publish the content specifically under the CC BY 4.0 license.

We recommend that you contact the original copyright holder with the Content Permission Form (http://journals.plos.org/plosone/s/file?id=7c09/content-permission-form.pdf) and the following text:

“I request permission for the open-access journal PLOS ONE to publish XXX under the Creative Commons Attribution License (CCAL) CC BY 4.0 (http://creativecommons.org/licenses/by/4.0/). Please be aware that this license allows unrestricted use and distribution, even commercially, by third parties. Please reply and provide explicit written permission to publish XXX under a CC BY license and complete the attached form.”

Please upload the completed Content Permission Form or other proof of granted permissions as an "Other" file with your submission.

In the figure caption of the copyrighted figure, please include the following text: “Reprinted from [ref] under a CC BY license, with permission from [name of publisher], original copyright [original copyright year].”

Authors' Response: Map of Khoy County extracted from Arc GIS Software. This is explained in caption of Figure 1.

2. If you are unable to obtain permission from the original copyright holder to publish these figures under the CC BY 4.0 license or if the copyright holder’s requirements are incompatible with the CC BY 4.0 license, please either i) remove the figure or ii) supply a replacement figure that complies with the CC BY 4.0 license. Please check copyright information on all replacement figures and update the figure caption with source information. If applicable, please specify in the figure caption text when a figure is similar but not identical to the original image and is therefore for illustrative purposes only.

The following resources for replacing copyrighted map figures may be helpful:

USGS National Map Viewer (public domain): http://viewer.nationalmap.gov/viewer/

The Gateway to Astronaut Photography of Earth (public domain): http://eol.jsc.nasa.gov/sseop/clickmap/

Maps at the CIA (public domain): https://www.cia.gov/library/publications/the-world-factbook/index.html and https://www.cia.gov/library/publications/cia-maps-publications/index.html

NASA Earth Observatory (public domain): http://earthobservatory.nasa.gov/

Landsat: http://landsat.visibleearth.nasa.gov/

USGS EROS (Earth Resources Observatory and Science (EROS) Center) (public domain): http://eros.usgs.gov/#

Natural Earth (public domain): http://www.naturalearthdata.com/

Authors' Response: Map of Khoy County extracted from Arc GIS Software. This is explained in caption of Figure 1.

5. We note you have included a table to which you do not refer in the text of your manuscript. Please ensure that you refer to Table 1 in your text; if accepted, production will need this reference to link the reader to the Table.

Authors' Response: Explanation about contents in Tables 1 and 2, starting from line 163.

6. If the reviewer comments include a recommendation to cite specific previously published works, please review and evaluate these publications to determine whether they are relevant and should be cited. There is no requirement to cite these works unless the editor has indicated otherwise.

Authors' Response: Thanks for reviewing.
